# Lipoma management with a minimally invasive 1,444 nm Nd:YAG laser technique

**DOI:** 10.3389/fmed.2022.1011468

**Published:** 2022-11-21

**Authors:** Domenico Piccolo, Mohammed Hussein Mutlag, Laura Pieri, Irene Fusco, Claudio Conforti, Giuliana Crisman, Paolo Bonan

**Affiliations:** ^1^Skin Centers, Avezzano, Italy; ^2^Roma Clinic, Baghdad, Iraq; ^3^El.En. Group, Calenzano, Italy; ^4^Department of Dermatology and Venereology, Dermatology Clinic, Maggiore Hospital, University of Trieste, Trieste, Italy; ^5^Laser Cutaneous Cosmetic and Plastic Surgery Unit, Villa Donatello Clinic, Florence, Italy

**Keywords:** lipoma, 1,444 nm Nd:YAG laser, subcutaneous intralesional application, liposuction, cellular disruption

## Abstract

**Background:**

Lipoma is the most common benign mesenchymal tumor that is composed of mature fat cells. Subdermal laser lipoma treatment may be recommended as an alternative to surgery for its removal.

**Purpose:**

The purpose of the study was to investigate the efficacy of the 1,444 nm Nd:YAG laser subcutaneous intralesional application as a treatment option for lipoma.

**Materials and methods:**

On 60 patients (37 women and 23 men) with lipomas localized above the muscle and lipomatosis in various regions, a subcutaneous, micro-pulsed 1,444 nm Nd:YAG laser procedure was executed. Before treatment, an ultrasound was performed and the lipomas were measured. The same lighting setup and photographic tools were used to take pictures of each patient.

**Results:**

The lipoma reduced or completely disappeared in all cases at the last follow-up, and no infections, burns, skin lesions, episodes of severe bleeding, or other serious adverse effects were reported. The most common transient side effects were ecchymosis and edema. Partial lesion reduction refers to rare cases of lipomatosis in which the lipomas were so small that suction and accurate positioning of the capsular membrane contours were impossible.

**Conclusion:**

Lipoma treatment with a 1,444 nm Nd:YAG laser is a safe and effective minimally invasive procedure without risk of scarring. For cellular disruption, laser treatment is an effective and safe option.

## Introduction

Lipomas are non-cancerous, fatty tissue growths that slowly develop under the skin. A round or oval-shaped mass of fat tissue that is easily moved when touched is referred to as a lipoma and usually does not cause pain. Lipomas are very common; approximately one in every 1,000 people has one, and they most commonly appear between the ages of 40 and 60, but can develop at any age. People of all genders can be affected, and they can even be present at birth; however, women are significantly more likely to be impacted than men. Lipomas can show anywhere in the body, although they are most frequently found in the back, torso, arms, legs, shoulders, neck, and forehead areas. If they press against a nerve or appear near a joint, they can be uncomfortable (1). Lipomas are all composed of white fat. Some lipomas contain blood vessels (angiolipoma, a painful lipoma) as well as other tissues such as fibrous tissue (fibrolipoma), brown fat (hibernoma), or tissue that produces blood cells (myelolipoma). To rule out dangerous disorders such as liposarcoma (a type of cancer), imaging tests like ultrasound, magnetic resonance imaging (MRI) scan, or a computed tomography (CT) scan must be used to analyze lipomas. The symptoms of liposarcoma are similar to those of lipoma. These benign soft tissue tumors can be removed with an outpatient procedure if they cause pain, grow to a large size, or grow in an uncomfortable area; Patients typically leave the hospital the same day after undergoing surgical lipoma excision; in fact, it is an effective and safe procedure. Liposuction may be recommended as an alternative to lipoma surgery to remove the lipoma; liposuction was actually introduced to achieve superior aesthetic results through the use of small incisions (2, 3). The first description of micropulsed Nd:YAG laser lipolysis with optical fiber was in 1994, and it is now one of the most widely used laser assisted lipoplasty methods in the world (4). Previous studies have demonstrated that pulsed Nd:YAG laser has lipolytic effects, causes the coagulation of small vessels and the reticular dermis, and the formation of neocollagen in the subcutaneous layer and dermal tissue. In the treatment of lipoma, Nd:YAG laser acts in two ways: As an optomechanical effect against the lipoma membrane and fat cells, and as thermal heating (thermal effect). The laser action fragments the capsular membrane of a lipoma in the same way that it fragments adipocyte membranes (5). Several interstitial laser systems have been developed over the years, with harmonic Nd:YAG wavelengths such as 1,064, 1,320, and 1,444 nm; additionally, the use of the Nd:YAG laser at 1,444 nm for the treatment of lipomas has been described in the literature. The 1,444 nm wavelength is very safe in laser-assisted lipolytic treatments because the energy is absorbed twice by both water and fat, keeping the tissue adjacent to the target area safe and protected. For laser-assisted lipolysis, it is even asserted that 1,444 nm wavelength is more efficient, because its affinity for fat is ten times that of an equivalent of 1,064 nm wavelength (6). Laser lipolysis is usually performed using a cannula containing a laser fiber and a minimally invasive approach; the small cannula used for laser irradiation is applied subcutaneously and moved back and forth to allow the laser energy to be adsorbed by the adipocytes. As a result, pores are formed in the adipocyte cell membranes, allowing dissolved fat to migrate into the extracellular space. Compared to traditional liposuction procedures, laser lipolysis causes less bleeding, bruising, and swelling, resulting in a faster recovery (4, 7, 8).

Based on literature findings, this study was conducted in order to assess the efficacy and safety of 1,444 nm Nd:YAG laser for lipoma management.

## Materials and methods

### Study device and patient population

A subcutaneous, micro-pulsed, 1,444 nm Nd:YAG laser procedure was used on sixty patients (median age, 39 years; range, 24–69 years; 37 women and 23 men) with lipomas localized above the muscle, and lipomatosis in various regions (LipoAI, Deka, Florence, Italy). The study device is intended for soft tissue surgical incision, excision, vaporization, ablation, and coagulation. Skin, cutaneous tissue, subcutaneous tissue, striated and smooth tissue, muscle, cartilage meniscus, mucous membrane, lymph vessels and nodes, organs, and glands are all included. The study device is also approved for laser-assisted lipolysis. Patients who were pregnant or lactating, as well as those with a history of keloid formation were excluded from the study.

### Study protocol

Before treatment, an ultrasound was performed and the lipomas were measured (max diameter 15 cm). Lesions were found on the face, neck, torso, and extremities. All patients were photographed in the same lighting setup for documentation. All subjects gave their informed consent. Before beginning the surgery, each lesion was identified and marked. Every process was carried out in an outpatient setting, under aseptic conditions, and subsequently an adequate subcutaneous local anaesthetic/tumescent solution was injected. To make the benign tumor stand out for easier removal, the anaesthetic solution was infiltrated into the tumor rather than the surrounding tissue. A cannula with a 600-m optical fiber was introduced through a 1 mm incision after making sure that the patient and the entire team had appropriate eye protection. The fiber passed through the proximal end of the clamp, in order for the fiber to be a little longer than the cannula. The fiber extended no more than 2–3 mm beyond the end of the cannula. When the laser emission was activated, the fiber tip had to be outside the cannula. At the end of the treatment, the handpiece was removed from the treatment area. To see the subcutaneous laser action while it was in use, the therapy beam and the targeting beam were coupled into the optical cable from the laser head. The risk of cutaneous burns and perforations was reduced by this trans-illumination effect, which gave the surgeon accurate knowledge of where and at what level the Nd:YAG laser was working. The more intense the aiming light, the more superficial (subdermal) the laser treatment. The following parameters were used to treat subjects in a single session of micropulsed (max 100 μs) subdermal 1,444 nm Nd:YAG laser energy: the pulse rate is 30 Hz, and the power was 6 W. The lipoma size and endpoint determined the total accumulated energy used in each case. The clinical endpoint was determined by palpation, the creation of the optimal contour and shape, and the elimination of cannula resistance, which triggered lipolysis and the subsequent transformation of the fatty tissue into an oily, less dense solution. The laser was applied to three layers in large lesions (more than 5 cm in largest diameter): deep, medium, and finally subdermal.

Using a 2-mm-diameter cannula and a negative pressure of 0.5 atm (50 kPa or 350–400 mmHg), an oily solution containing fat cell debris and membrane dissolved lipid was aspirated from the area after treatment. The incision site was closed with sutures or covered with sterile-strips; at the end of the procedure, it was covered with a smooth, non-adherent antimicrobial dressing, such as compression bandages, for 1-week post procedure. Following surgery, the patients were checked on a regular basis. The follow-up period ranged from 1 week to 6 months, with 1- and 3-month interim visits.

### Adverse events

During the study all possible adverse effects at the treatment site were monitored.

## Results

**FIGURE 1:**
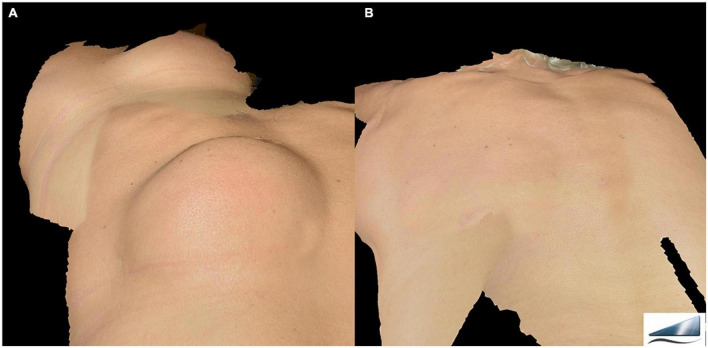
Image of giant lipoma placed on patient’s back acquired using the 3D Photography System LifeViz^®^ Mini (QuantifiCare) before **(A)** and 6 months after laser treatment **(B)**. A complete removal of the lipoma is observed without any aesthetic damage.

**FIGURE 2:**
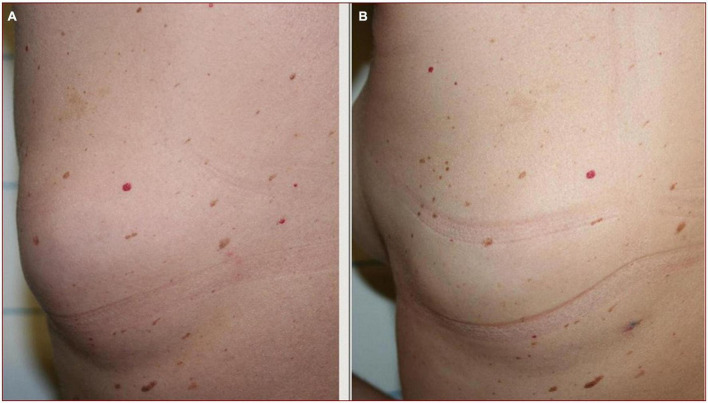
Lipoma on patient’s back **(A)**. Photographic evaluation shows a complete lipoma removal after laser treatment **(B)**.

**FIGURE 3:**
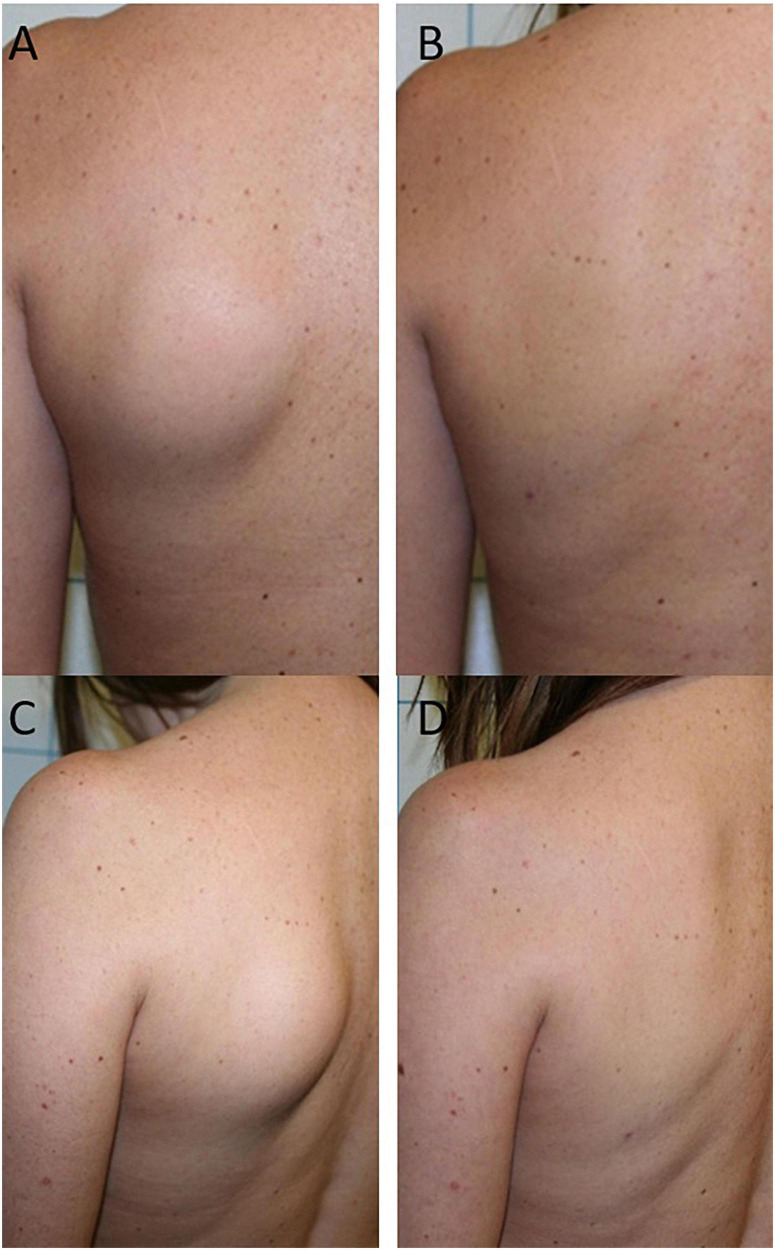
Frontal **(A)** and lateral **(C)** view of the lipoma located on the left side of the patient’s back. A complete removal of the lipoma is observed 1 month after laser treatment without leaving visible aesthetic marks **(B,D)**.

All patients were pleased with the small 1 mm wounds from the cannula entry point well, which were no longer visible a few months after laser treatment. Patients returned for follow-up exams on a regular basis following laser surgery. At the last follow-up, the lipoma had reduced or completely disappeared in all cases, and no infections, burns, skin lesions, episodes of severe bleeding, or other serious adverse effects were reported. Two cases representing the aesthetic improvement of the lipoma after treatment are clearly shown in Figures 1–3. The transient side effects were mostly ecchymosis and edema. All subjects tolerated the postsurgical period well. Patients generally reported a “prickling” sensation for a couple of days without severe pain. Cases of partial lesion reduction refer to rare cases of lipomatosis in which the dimensions of the lipomas were so small that suction and accurate positioning of the contours of the capsular membrane were impossible.

## Discussion

A lipoma is a benign soft tissue tumor composed of fat cells that grows and often requires removal as the benign tumor grows because it can become not only an aesthetic problem but can also cause nerve compression. Although the exact mechanism of lipoma formation is uncertain, it has been suggested that following blunt trauma, rupture of the fibrous septum may result in adipose tissue proliferation (9). Surgery and liposuction have been the typical treatments for this condition, allowing for histopathological analysis of the tissue removed. The main disadvantage of surgical excision is the possibility of large and visible scars that will be permanent due to the size of the tumor and the possible recovery time. Liposuction has been suggested as a less invasive and more aesthetically pleasing treatment option (10, 11). Although liposuction is widely used, the presence of fibrous structures in lipomas and the small dimensions of benign tumors can be a limitation in some cases; in fact, relapse rates are higher in these cases because the remains of the capsule can be left in place. Liposuction therapy is not appropriate for the rare CD34-positive spindle cell lipoma, which can be “fat-free” (12). It has been reported in the literature that up to 10% of lipomas are not completely removed (11). The use of laser therapy for the treatment of lipomas is already validated in the literature (Table 1).

**TABLE 1:** Clinical studies using non-ablative and ablative lasers for lipomas.

References	Laser	Patient number	Lipoma type	Methods	Findings
Stebbins et al. (20)	980 nm diode laser	1	Back lipoma	Laser lipolysis was performed using a continuous wave. Laser energy was delivered to the lipoma *via* a 600-mm fiberoptic ensheathed in a 1.2-mm diameter stainless steel cannula, with the distal 2 mm of the fiber extending beyond the distal tip of the cannula. The power was set at 20 W, and a total of 7,000 J were administered to the lipoma. Total treatment time was approximately 20 min.	No evidence of lipoma on U/S at 2-months follow-up. 90% reduction on U/S at 1-month follow-up.
Min Lee et al. (21)	1,444-nm neodymium-doped yttrium aluminum garnet (Nd:YAG)	3	Back lipoma	Subjects were treated in a single session of micropulsed subdermal 1,444-nm Nd:YAG laser energy using the following parameters: pulse rate 30 Hz, pulse energy 200 mJ, pulse width 100 ms, power 6 W. The total accumulated energy used in each case ranged from 1,100 to 4,200 J/cm^2^ and was determined by the lipoma size.	In all cases, reduction or complete disappearance of the lipoma was observed at 6-month follow-up, and no infections, episodes of severe bleeding, or any other serious adverse effects were reported.
Goldman and Wollina (1)	1,064-nm Nd:YAG	20 patients (11 women and 9 men)	Subdermal lipoma	The laser energy was delivered to the subcutaneous tissue in direct contact with the lipoma through a 300-lm fiberoptic with a 1-mm-diameter stainless steel microcannula of variable length connected to the tip of the fiber. In large lipomas (more than 5 cm in diameter), a 600-μm fiberoptic with a 1.2-mm-diameter cannula was used. The total accumulated energy used in each lesion ranged from 5,000 to 32,000 J/cm^2^.	Subdermal lipoma treatment using a 1,064-nm Nd:YAG laser resulted in complete or almost complete removal of the tumor in 100% of patients. Four partial relapses were observed that were treated successfully by the same procedure. Adverse effects were mild and temporary.
Sergey et al. (22)	960 nm diode laser	1	Nasopharyngeal lipoma	Neoplasm was removed by using an 8 W contact diode laser with 0° rigid endoscope, under topical anesthesia with 2 ml of 10% lidocaine solution.	The endoscopic endonasal approach with the use of a 960 nm diode laser and topical anesthesia was implemented to remove the neoplasm non-invasively and thus achieve a good long-term result.
Lombardo et al. (23)	CO_2_ laser	1	Laryngeal lipoma	The dimension of the lesion with its well-defined capsule edging allowed us to perform an excisional biopsy using TLM with CO_2_ laser; in addition, we made a continuous wave laser treatment on the excisional margins and to the wound bed to avoid relapses.	The use of transoral laser CO_2_ micro-laryngoscopy (TLM) provided good management of a small intrinsic lipomas of the larynx, minimizing the potential for relapses.

A subdermal Nd:YAG laser method was used to treat 83.3% of lesions in a single session. The side effects were minor and transient, and the small 1–2 mm wound was well tolerated by all patients (1). The recurrence rate in giant lipomas was higher, approaching 18% in surgically removed tumors (13). Indeed, giant lipomas are therapeutically challenging (14, 15), and in these cases, the ability to prevent lipoma relapse using the same minimally invasive 1,444 nm device and technique, while avoiding large scars, is advantageous. The use of the pulsed 1,444 nm Nd:YAG laser as a treatment option for lipomas overcomes the limitations of traditional tumescent liposuction with minimal invasiveness. Due to the excellent aesthetic results, the need for multiple treatments does not appear to be a major disadvantage in some patients. Compared to the 1,064 nm wavelength, the 1,444 nm wavelength has a substantially higher duality of absorption in both water and fat (16). The high fat absorption ensures efficient lipolysis, and the high-water absorption confines the thermal reaction to the tissues surrounding the optical fiber tip. In *in vivo* minipig and *in vitro* human fat experiments, the 1,444 nm Nd:YAG laser demonstrated a greater lipolytic effect than the 1,064 nm Nd:YAG laser (6). Since its initial description in 1994, Nd:YAG laser lipolysis has mostly been used in Europe and South America (4). It is now one of the most widely used laser lipoplasty techniques worldwide. Laser lipolysis is usually performed with a cannula containing a laser fiber that is inserted into the treatment area. To allow the laser energy to dissolve excess fat, the cannula is moved back and forth. The photoacoustic effect mechanically disrupts adipocytes, whereas the photothermal effect converts laser light into heat energy in fat, collagenous tissue, and hemoglobin. Heat causes increased liquefaction and disruption of the cell membrane, allowing for extracellular drainage. The heat causes coagulation of small vessels in the fat tissues, which makes the procedure easier by reducing trauma and bleeding and allowing for faster recovery. The heating of the deep dermis and conjunctive septa of subcutaneous tissues causes collagen denaturation, which is followed by vascular proliferation and collagen neosynthesis. According to a recent study, 1,064 and 1,320 nm have collagen as the primary tissue target, with adipocyte damage occurring secondarily (8). Furthermore, when compared to the other two wavelengths for laser-assisted lipolysis, 1,320 and 1,064 nm, 1,444 nm have the highest ablation efficiency with the least amount of heat localization over the depth (17). Since its introduction, numerous publications have claimed that laser-assisted lipolysis is significantly better than conventional liposuction. Katz et al. suggested that conventional liposuction may worsen skin laxity (18), but Min et al. propose that, in addition to its well-known indications for liposculpture of the face and body, therapy with the 1,444-nm Nd:YAG laser may also be utilized for skin tightening. As a result, the 1,444 nm laser may enhance the beneficial effects of laser lipolysis, such as dermal tightening, less bleeding and pain, minimal tissue damage, and faster recovery (19).

### Study limitations

One of the limitations of the study is the absence of a patient’s satisfaction assessment. Our future goal will be to investigate the indicative times and the patient satisfaction index, in order to better validate our scientific findings.

## Conclusion

Our findings showed that lipoma treatment with a 1,444 nm Nd:YAG laser is a minimally invasive, scar-free, safe, and effective procedure.

## Data availability statement

The original contributions presented in the study are included in the article, further inquiries can be directed to the corresponding author.

## Ethics statement

Ethical review and approval was not required for the study on human participants in accordance with the local legislation and institutional requirements. Written informed consent was obtained from the individual(s) for the publication of any identifiable images or data included in this article.

## Author contributions

DP, MM, LP, CC, GC, and PB: conceptualization, methodology, validation, supervision, and funding acquisition. LP: formal analysis and writing—original draft preparation. LP, MM, and IF: investigation. DP, MM, LP, CC, and GC: data curation. LP and IF: writing—review and editing. All authors have read and agreed to the published version of the manuscript.
